# A Systematic Review of the Short-Term Health Effects of Air Pollution in Persons Living with Coronary Heart Disease

**DOI:** 10.3390/jcm8020274

**Published:** 2019-02-24

**Authors:** Darren E. R. Warburton, Shannon S. D. Bredin, Erin M. Shellington, Christie Cole, Amanda de Faye, Jennifer Harris, David D. Kim, Alan Abelsohn

**Affiliations:** 1 Physical Activity Promotion and Chronic Disease Prevention Unit, University of British Columbia, Vancouver, BC V6T 1Z4, Canada; shannon.bredin@ubc.ca (S.S.D.B.); erin.shellington@ubc.ca (E.M.S.); amanda_defaye@hotmail.com (A.d.F.); davidd.kim@alumni.ubc.ca (D.D.K.); 2 Laboratory for Knowledge Mobilization, University of British Columbia, Vancouver, BC V6T 1Z4, Canada; 3 Department of Heart Wise Exercise, University of Ottawa Heart Institute, Ottawa, ON K1Y 4W7, Canada; cacole@ottawaheart.ca (C.C.); jharris@ottawaheart.ca (J.H.); 4 Department of Family and Community Medicine and Dalla Lana School of Public Health, University of Toronto, Toronto, ON M5S 1A1, Canada; alan.abelsohn@utoronto.ca

**Keywords:** exercise, air quality health index, coronary artery disease

## Abstract

Persons living with chronic medical conditions (such as coronary artery disease (CAD)) are thought to be at increased risk when exposed to air pollution. This systematic review critically evaluated the short-term health effects of air pollution in persons living with CAD. Original research articles were retrieved systematically through searching electronic databases (e.g., Medical Literature Analysis and Retrieval System Online (MEDLINE)), cross-referencing, and the authors’ knowledge. From 2884 individual citations, 26 eligible articles were identified. The majority of the investigations (18 of 22 (82%)) revealed a negative relationship between air pollutants and cardiac function or overall health. Heart rate variability (HRV) was the primary cardiovascular outcome measure, with 10 out of 13 studies reporting at least one index of HRV being significantly affected by air pollutants. However, there was some inconsistency in the relationship between HRV and air pollutants, mediated (at least in part) by the confounding effects of beta-blocker medications. In conclusion, there is strong evidence that air pollution can have adverse effects on cardiovascular function in persons living with CAD. All persons living with CAD should be educated on how to monitor air quality, should recognize the potential risks of excessive exposure to air pollution, and be aware of strategies to mitigate these risks. Persons living with CAD should minimize their exposure to air pollution by limiting outdoor physical activity participation when the forecast air quality health index indicates increased air pollution (i.e., an increased risk).

## 1. Introduction

Air pollution has been associated with various health issues, including worsening of heart and pulmonary conditions and increased risk for asthma, heart attacks, stroke, and premature mortality [1,2,3]. The World Health Organization estimates that ambient air pollution contributes to 6.7% of all deaths [4]. In 2008, the Canadian Medical Association estimated that approximately 11,000 hospital admissions, 92,000 emergency department visits, and 21,000 deaths annually could be attributed to air pollution [5]. 

Particulate matter (PM) is the most common air pollution associated with adverse health effects [6], and is often defined as coarse (2.5 to 10 μm (PM_10–2.5_)), fine (≤2.5 μm (PM_2.5_)), or ultrafine (≤0.1 μm (PM_0.1_)) particles [7,8,9]. Gaseous pollutants (such as nitrogen dioxide (NO_2_), ozone, sulphur dioxide (SO_2_), and carbon monoxide (CO)) have also been consistently related to adverse health outcomes [1,6,10]. In Canada, the Air Quality Health Index (AQHI) considers the presence of three primary air pollutants (i.e., ozone, fine particular matter (PM_2.5_), and NO_2_) to estimate health risk [11].

Although ambient air pollutant concentrations appear to be decreasing overall [12], the elderly, children, pregnant women, those living with chronic medical conditions, and those with weakened immune systems appear to be particularly vulnerable to air pollution exposure and require special precautions [6,13,14,15,16]. Specifically, in persons living with coronary artery disease (CAD), air pollution has been associated with various complications such as increased cardiovascular disease-related hospitalizations/readmissions and premature mortality [1,10]. Several mechanisms have been associated with increased risk, including changes in blood viscosity, oxidative stress and inflammation, vascular dysfunction, and altered autonomic function (such as decreased heart rate variability (HRV)) [6,17]. The majority of the research relates to the long-term effects of air pollution exposure; however, mounting research has demonstrated the short-term adverse effects of air pollution [7,9,18,19,20,21,22,23,24]. 

Routine exercise and/or physical activity participation is key to the secondary prevention of CAD [25]. However, it remains unclear when the benefits of physical activity might be outweighed by the short-term risks associated with exercising in a polluted environment. A clearer understanding of the short-term risks associated with air pollution will provide further insight for optimum exercise recommendations when considering ambient air pollution for this sub-population and help inform recommendations made by air quality indexes [11,26].

To our knowledge, no systematic review has outlined the short-term health effects of air pollution in persons living with CAD. Accordingly, the primary objective was to critically examine the current literature to determine the short-term health risks associated with air pollution in persons living with CAD. A further objective was to provide recommendations to mediate these risks in patients with CAD in the context of the standard of care for secondary prevention including whether alterations in physical activity behaviour are warranted based on air pollution levels. 

## 2. Experimental Section

### 2.1. Criteria for Considering Studies for this Review

A rigorous, systematic, and evidence-based approach was used to examine the evidence regarding the short-term health effects of air pollution in persons living with CAD. We adhered to the standards established by the Preferred Reporting Items for Systematic Reviews and Meta-Analyses (PRISMA) recommendations [27]. For inclusion, studies needed to define clearly the exposure of air pollution, provide documented patient history of CAD, and have a clearly articulated cardiovascular outcome measure. All original studies that evaluated the effects of occupational exposures or reported multiple conditions besides CAD without a sub-group analysis were excluded.

### 2.2. Search Strategy 

Literature searches were conducted in the following electronic bibliographical databases (Table 1):

- Medical Literature Analysis and Retrieval System Online (MEDLINE; 1948–January 2019, Ovid Technologies, Inc. (Ovid) Interface, New York, NY, USA)
- Excerpta Medica database (EMBASE) (1980–January 2019, Ovid Interface, New York, NY, USA)
- Cochrane Library (1994–January 2019, Ovid Interface, New York, NY, USA)

### 2.3. Screening

Two reviewers independently screened titles and abstracts of citations to identify potential articles for inclusion and to remove duplicate citations [28]. Full-text versions of relevant articles were obtained, and data was extracted with a common template. In the case of disagreement (5% of cases), a third reviewer was included in order to achieve a full (100%) consensus. Reasons for exclusion of articles after full-article screening were recorded [28,29].

Two reviewers independently evaluated the level, grade, and quality, with consensus achieved through discussion as required (Table 2). The quality of investigations was assessed with the Downs and Black scoring system [30] as modified previously [29]. 

## 3. Results

### 3.1. Overview of Studies Selected

**Figure 1:**
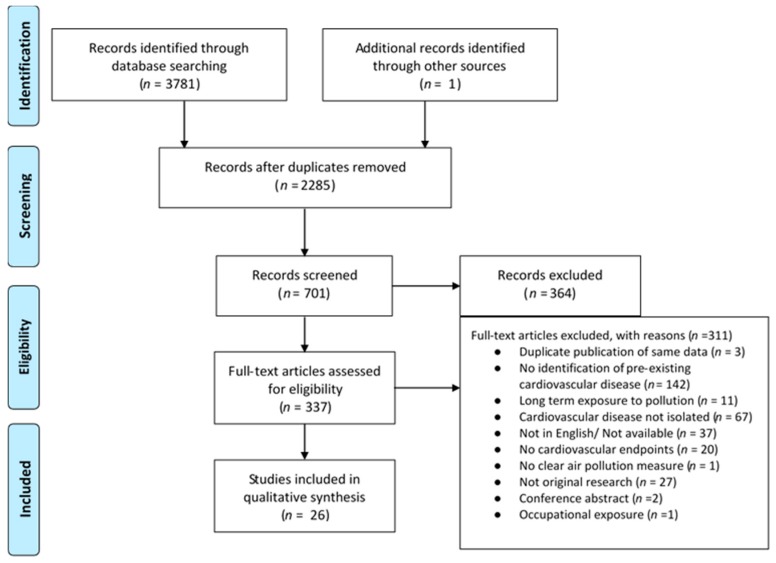
Results of the literature search for air quality and exercise in persons living with coronary artery disease; *n* is an abbreviation for number.

A total of 26 articles were included in the final systematic review (Table 3 and Table 4, Figure 1) including seven laboratory investigations and 19 real-life setting investigations, nine of which examined adverse effects during exercise. Quality assessment of articles varied considerably, but generally produced medium-quality investigations, with scores ranging from 8 to 13 out of 15 (Mean = 9.7 ± 1.3).

During the laboratory trials, exposure to various air pollutants were evaluated for adverse effects including ST-segment depressions, angina, arterial carboxyhemoglobin levels, arrhythmias, measures of HRV, heart rate (HR), and blood pressure (BP) [31,32,33,34,35,36], central aortic pulse wave velocity and augmentation index [37]. Two studies looked at ST-segment depression related to pollution exposures in lab [31,32], while four studies considered exposures in real-life settings [37,38,39,40]. Adverse associations were found between traffic exposure with exercise and subsequent ST-segment depression with CO in patients with CAD and survivors of myocardial infarction (MI) [31,32]. Ambient PM_1_ [39], PM_2.5_ [39,40], number counts of particles sized 0.1–1 μm [39], ultrafine particles sized 0.01–0.1 μm [38], black carbon [39], CO [38], diesel exhaust [32], and SO_2_ [39] were also associated with increased ST depression in patients with CAD. One study additionally reported expedited development of exercise-induced angina as well as increased mean HR after exposure to freeway air [31], though another found no difference in HR during exposures to filtered air and diesel exhaust [32]. Lastly, a recent study found that the vascular benefits associated with exercise were attenuated in a polluted street compared to a non-polluted urban park in adults with ischemic heart disease [37].

### 3.2. Blood Markers and Heart Rhythm 

Two laboratory studies reported significantly increased arterial carboxyhemoglobin and expired air CO levels after exposure to heavy traffic exhaust or CO [31,33]. Ventricular arrhythmias were studied in conjunction with 4% and 6% mean carboxyhemoglobin levels [33]. These studies revealed that exposure to 200 ppm of CO induced 6% carboxyhemoglobin levels in CAD patients, which was associated with increased number and complexity of ventricular arrhythmias. 

One retrospective study analyzed electrocardiograms (ECGs) from a database of patients with coronary artery disease and correlated ECG abnormalities with centrally monitored PM_2.5_ and ozone for up to four days prior to ECG recordings; it was found that increased air pollutants (ozone and PM_2.5_) were associated with lengthened PR, QRS, and QT intervals and increased heart rate resulting in ECG abnormalities for up to seven days [44].

### 3.3. Vascular Measures

Blood pressure and HR were examined as secondary measures in five investigations [31,32,34,35,37]. Results from two studies conflicted with others, finding systolic BP and HR is negatively associated with heavy traffic in patients living with CAD whereas the other studies showed no relationship [31,32,33,35]. A recent study found an adverse association between walking in a polluted area (Oxford Street, London, UK) compared to a non-polluted area (Hyde Park, London, UK), whereby the polluted area (black carbon, PM_2.5_, PM_10_, and ultrafine particles) impaired benefits on pulse wave velocity and augmentation index following an acute bout of exercise in adults with ischemic heart disease [37]. Lastly, a study found that decreased large artery elasticity index was associated with increasing ozone in adults with coronary artery disease [41].

### 3.4. Heart Rate Variability Outcomes

Three laboratory studies investigated HRV after exposure to NO_2_, diesel exhaust, SO_2_, or carbon particles [34,35,36]. None of these studies found an association between HRV indices and the various pollutants in patients with stable angina or CAD. Twelve real-life studies identified reduced HRV measures associated with exposures to PM_10_ [38], PM_10–2.5_ [38], PM_2.5_ [19,24,45], PM_0.3–1.0_ [7], SO_2_ [34], NO_2_ [20,35], CO [18,21,24,45], elemental carbon [20], and black carbon [45]. Reduced standard deviation of normal-to-normal (N-N) intervals (SDNN) was observed in a number of studies, associated with PM of different sizes as well as CO, and black carbon exposure [7,18,19,38,45]. The length of time between the exposure and observed adverse HRV effects varied greatly from a short lag period of 2 h or less for black carbon [45], to 2- and 3-day lag periods for PM_2.5_ [19]. A decrease in the root mean square of successive differences between N-N intervals (rMSSD) was frequently found among HRV results. Multiple air pollutants (PM_2.5_, PM_0.3–1.0_, NO_2_, CO, black carbon, and elemental carbon) were found to reduce rMSSD in the CAD population, over timelines that varied in effect from 30 min to 24 h [7,20,21,45]. Decreases in the high frequency (HF) index of HRV were frequently explored in these investigations, with PM_2.5_ being associated with these reductions at time scales extending from 30 min through 5 days [19,24,45]. Reduced HF was also associated with increases in elemental carbon [20], black carbon [45], and NO_2_ [20] in individual study examples. Participants with CAD in a cardiac rehabilitation program showed an association between decreased rMSSD and accumulation mode particles (AMP; diameter 100–1000 nm) in a 6–24 h lag period, and non-significant decreases in rMSSD were associated with ultrafine particles and PM_2.5_ prior to exercise. In addition, increased systolic blood pressure (0.84 mmHg) was significantly associated with ultrafine particles, AMP, and PM_2.5_ for 0–24 h lag periods in the pre-exercise period; no significant associations were found in SDNN or mean N-N and no significant associations were found during the exercise sessions in HRV [42]. 

Conversely, some studies showed no differences in HRV when exposed to air pollutants. Specifically, SO_2_ showed no impact on patients with stable angina 4 h after a 1-h exposure [34], and exposure to PM_2.5–10_ [7] and PM_1.0–2.5_ [7], PM_2.5_ [9,18,20,22], NO_2_ [35], diesel exhaust [35], and elemental carbon [20] and ozone [41] did not impact HRV. 

Other indices of HRV were explored in single studies only, and in some cases the HRV indices are controversial in interpretation. Importantly, a few studies mentioned sub-analyses that demonstrated differences in HRV indices when comparing sub-groups that used different medications such as beta-blockers, and in a few cases the study included a majority of patients taking beta-blocking agents [24,32,33,34,36,45] without providing a specific sub-analysis. In some papers, beta-blocking agents appeared to attenuate the adverse effects of air pollution exposure to patients [19,38]. This aligned well with papers stating patients taking beta-blockers did not share the same associations with decreased HRV indices and increased air pollutants [18]. Other commonly used medications such as calcium channel blockers or statins were frequently mentioned in the descriptive statistics; however, often no sub-analyses were provided to further explore the possibility of effect modification, in part due to insufficient power for proper analysis. One study did disclose that patients with diabetes experienced more substantial reductions in rMSSD compared to participants without diabetes [23]. 

### 3.5. Morbidity and Mortality Outcomes 

In real-life setting studies of CAD patients, a single study identified that daily non-trauma mortality was increased when particle number concentration and PM_10_ averages over 2 days were increased [6]. Averaging times of 5 and 15 days for CO and NO_2_ were associated with mortality as well [6]; however, this was the only study that considered mortality in patients with confirmed CAD. Another study revealed that short-term ozone exposure (within 1–2 days) was related to acute myocardial infarction in middle-aged adults without heart disease [43]. However, no associations were found between acute myocardial infarction in participants with a history of CAD after exposure to elevated levels of ozone, NO_2_, or SO_2_. No other environmental studies identified mortality as an endpoint in patients previously diagnosed with CAD; however, one group identified increased same-day readmissions for MI survivors when concentrations of many pollutants were elevated [10].

## 4. Discussion

While the negative long-term health effects of air pollution are well established (i.e., increased morbidity and premature mortality), it is unclear whether individuals living with CAD are more susceptible to short-term adverse cardiovascular events than the general population. To the best of our knowledge, this systematic review is the first to evaluate this subject. Inconsistent findings regarding the effects of various air pollutants on cardiovascular endpoints were identified through this review; however, the majority of the studies revealed that short-term exposure to air pollution is associated with adverse cardiovascular events and disturbances in autonomic function in persons living with CAD. The adverse effects were dependent on the type of pollutant, the site of exposure measurement (personal versus ambient), and whether patients were taking beta-blocker medication [18,19,20]. 

### 4.1. Short-Term Risks of Air Pollution

There is considerable research demonstrating the long-term effects of exposure to air pollution on the risk for cardiovascular-related adverse events and premature mortality [45,46]. For instance, the Harvard Six Cities study revealed that air pollution was directly associated with premature mortality from lung cancer and cardiopulmonary disease in 8111 adults (14 to 16-year follow-up) [47]. A meta-analysis revealed that the risk for all-cause mortality was increased 0.6% (95% confidence interval, CI = 0.5–0.7) per 10 μg/m^3^ elevation in PM with an aerodynamic diameter of PM_10_ [48].

As outlined in this systematic review there is also compelling evidence documenting the short-term risks associated with air pollution in persons living with CAD. Research suggests that fine particles may have the greatest cardiovascular effects [45]. The most commonly studied cardiovascular outcome measure in our current systematic review was HRV, a widely used non-invasive measurement of cardiovascular autonomic control [21] associated with the risk for adverse cardiovascular events, sudden cardiac death, and premature all-cause mortality [49]. Compromised cardiovascular autonomic control is thought to be a mechanism between ambient air pollution and cardiovascular mortality [19,21,50]. In the current systematic review, HRV was found to be negatively associated with multiple pollutants including black carbon, carbon dioxide, CO, and NO_2_ as well as mass concentrations of PM_0.3–1.0_, PM_2.5_, PM_2.5–10_, PM_10_, and AMP [7,9,18,19,20,21,22,23,24,42]. Similarly, air pollution was associated with short-term risks for adverse cardiovascular events including cardiac readmissions, myocardial ischemia, angina, increased number and complexity of ventricular arrhythmias, and increased all-cause mortality [6,10,31,32,33]. Furthermore, ST-segment depression (as an indicator of myocardial ischemia) was the most common index of adverse cardiovascular effects of air pollution exposure (next to HRV). These studies revealed that ST-segment depression was associated with diesel exhaust, black carbon, and particulate air pollution [31,32,39,40] in addition to other ECG abnormalities (lengthened PR, QRS, and intervals) associated with ozone and PM_2.5_ [44]. Lanki and colleagues concluded that particulate air pollution originating from combustion processes (particularly traffic) exacerbated ischemic heart disease in persons living with CAD [40]. Delfino and colleagues [17] also reported that exposure to organic carbon was associated with a significant increase in systolic (8.2 mmHg) and diastolic (5.8 mmHg) BP in elderly participants with CAD. Rich et al., (2012) reported ultrafine particles, PM_2.5_ and AMP were associated with increased systolic blood pressure (0.94 mmHg) [42] and Sinharay et al. (2018) reported air pollution impaired benefits of exercise compared to clear air environments in adults with CAD [37]. Collectively, these findings indicate that short-term air pollution exposure increases the risk of adverse cardiovascular events and disturbances in autonomic function in persons living with CAD. Moreover, this population appears to be at increased risk for adverse cardiovascular events related to air pollution. 

Patients living with CAD often live with hypertension as well. However, few studies have examined the effects of air pollution on populations with coexisting hypertension and CAD. A recent study [51] revealed greater air pollution-related risks in CAD patients with pre-existing hypertension (odds ratio (OR) = 1.39; 95% CI = 1.10, 1.76) compared to normotensive patients (OR = 0.90; 95% CI = 0.66, 1.23). This preliminary research (and that of others included in this systematic review [34,36]) indicates that patients living with CAD and hypertension should pay particular attention to air pollution exposure and take appropriate steps to mitigate risks.

Beta-blocker medication likely moderates the interaction between short-term exposure to air pollution and cardiovascular function. Previous research has found that beta-blocker medication increases cardiac vagal control, which may explain the reported inconsistencies in the literature [19,36,52,53]. Multiple studies in this review revealed statistically greater effects of exposure on cardiovascular function in patients who were not taking beta-blocker medication compared to those who were [18,19,38]. For instance, Dales and colleagues [18] reported that patients not taking beta-blocker medication had significantly decreased indices of HRV after exposure to high levels of CO, but patients on beta-blockers showed no effects. Three of the four studies that reported no effect of air pollution on CAD patients attributed their findings to beta-blocker medication [34,36,43]. These findings provide compelling evidence that beta-blocker medications may mask the cardiac effects that air pollutants have on individuals living with CAD.

### 4.2. Air Pollution and Exercise in Persons Living with CAD 

The health benefits of routine exercise and physical activity participation for persons living with CAD are irrefutable [25]. Cardiac rehabilitation is considered a global “standard of care” for the treatment of CAD [54] and has been associated with marked reductions in hospitalizations/readmissions, and premature cardiac- and all-cause mortality [55]. Risk reductions of 25–30% for premature cardiac-related mortality have been consistently observed in cardiac rehabilitation trials including both home- and centre-based trials [55]. The most effective means of exercise rehabilitation have been refined throughout the years, more recently adhering to evidence-based best practice guidelines related to effective pre-participation screening, risk stratification, behaviour modification, and exercise prescription for persons living with CAD [56,57]. 

Air quality indicators are in use across in North America that provide recommendations to the general public regarding the risks associated with air pollution. In Canada, the AQHI is a publicly available tool that outlines the daily health risks associated with air pollution across Canada on a scale from 1 to 10 (i.e., very low to very high health risk, respectively) (Supplementary Figure S1). It provides advice about appropriate modification of outdoor physical activities during increased levels of risk, both for the general population and for those most affected by air pollution (such as people with heart and lung conditions). A similar approach has been taken in the United States with the Air Quality Index (AQI). These strategies are important examples of knowledge translation of evidence to the general public. Unfortunately, their success has been limited [58] and inclusion of recommendations related to air pollution does not occur systematically in cardiac rehabilitation settings. This is particularly important since the effectiveness and/or safety of exercise may be compromised when persons living with CAD exercise in polluted environments. 

Several studies have indicated increased myocardial ischemia during exercise when persons living with CAD are exposed to air pollution [32,38,40]. For instance, Lanki and colleagues [40] revealed that PM_2.5_ was associated with ST segment depression during submaximal exercise testing in stable CAD patients. Similarly, brief exposure to diluted diesel exhaust exacerbated myocardial ischemia (during exercise) and inhibited endogenous fibrinolytic capacity in MI patients [32]. Pekkanen and colleagues [38] reported independent associations of PM_2.5_ and ultrafine particulate air pollution and ST-segment depression during repeat exercise tests in individuals living with CAD. Other adverse cardiovascular symptoms have also been demonstrated during exercise after exposure to air pollution including an earlier onset of anginal symptoms [31], and increased number, complexity, and severity of ventricular arrhythmias [33], pulse wave velocity, and augmentation index [37].

Evidence-based best practice suggests that healthcare and exercise professionals working in cardiac rehabilitation settings and/or prescribing exercise to patients with CAD should be educated themselves and be educating patients about the potential increased risk associated with exercising in more polluted environments and how to mitigate the risks associated with air pollution using air quality indicators tools such as the AQI and AQHI.

### 4.3. Limitations

Many of the studies that were included in this review had small sample sizes and had varied air pollution exposures and cardiovascular outcome measures. As such, there are limitations to the conclusions we can make based upon the currently published literature in the area of short-term exposure to air pollution on the cardiovascular health in adults living with CAD. The small sample sizes may make overt generalizations difficult. However, the findings from this study included patients with CAD from diverse settings and countries. It is anticipated that these findings have strong applicability to those living with CAD. 

Additionally, limitations exist on the primary cardiovascular outcome measures that were used within the selected studies. Although most studies employed some form of control, heart rate variability can be affected by medication usage, fitness level, age, co-morbid conditions, and measurement conditions (e.g., exposures to caffeine or alcohol prior to measurement). This is true of other cardiovascular outcome measurements as well (e.g., arterial stiffness and blood pressure). We acknowledge that short-term exposures to air pollution do not necessarily translate/extrapolate to long-term effects. However, given that persons living with CAD are recommended to exercise, (over multiple acute bouts) our current findings demonstrate the potential cardiovascular risks associated with exercising in polluted areas and therefore, should be taken into consideration. 

### 4.4. Recommendations

A series of key recommendations emanate from our systematic review of the literature:

(1) Persons living with CAD are at an increased risk for short-term adverse cardiovascular-related events (such as strokes and exacerbation of pre-existing cardiovascular disease) and disturbances in autonomic function when exposed to air pollution in comparison to healthy individuals (1A).
(2) All qualified exercise professionals and healthcare providers should be aware of the increased risks associated with exercising in polluted environments in healthy individuals and persons living with chronic medical conditions (such as CAD) (4C).
(3) Persons living with CAD should carefully monitor air quality indicators (such as the AQI and AQHI) and exercise when air pollution is lower. These cautions are given with the understanding that the health benefits of routine physical activity are marked for persons living with CAD (1A).
(4) Qualified exercise professionals and healthcare providers working within cardiac rehabilitation settings should monitor air quality indicators (such as the AQHI) and caution patients living with CAD about the short-term risks of exercising in a polluted environment. This warning should be given with the understanding that the health benefits of routine physical activity are marked for persons living with CAD (4C).
(5) Persons living with CAD should avoid exercising in areas of high air pollution (such as near high-traffic areas, factories, or during wildfires) (1A).

### 4.5. Future Directions

There was variability in the type of exposure and cardiovascular measurement in the studies selected, which did not allow for a meta-analysis to assess the size of the effect. However, there is strong evidence that short-term exposure to air pollution carries an increased risk for adverse cardiovascular events, particularly during exercise. Further research is required to determine the magnitude of the risk, and how to best prescribe exercise and activities while avoiding air pollutants that carry the greatest risk for those living with CAD.

## 5. Conclusions

The current systematic review of the literature provides strong evidence that persons living with CAD are at an increased risk for short-term adverse cardiovascular-related events when exposed to air pollution. These adverse risks occur during exercise in polluted environments and may be altered by beta-blocker usage. As such, persons living with CAD and their healthcare providers (including qualified exercise professionals) should carefully monitor air quality indicators (such as the AQI and AQHI) and take precautions. This includes exercising indoors when air pollution risks are higher or rescheduling outdoor exercise to when air pollution risks are lower, as well as avoiding areas of high air pollution, especially near high-traffic areas or during air pollution events such as forest fires.

## Figures and Tables

**Table 1:** Results of the literature search regarding air pollution and health effects in persons living with coronary artery disease.

#	Search (January 2019)	MEDLINE	EMBASE	COCHRANE
1	heart rate/or urban health/or ischemic heart disease/or coronary artery disease/or coronary disease/or heart disease	621,135	1,438,474	53,299
2	air pollution/or air pollutant/or environmental exposure/or particle size/or particulate matter	66,211	324,479	2438
3	1 and 2	3159	8361	159
4	Limit 3 to English, humans, and adults < 18–65+ years	1492	2170	149

Notes: # represents database search result number; MEDLINE: Medical Literature Analysis and Retrieval System Online; EMBASE: Excerpta Medica database; COCHRANE: Cochrane Library.

**Table 2:** Level and grade of evidence criteria for the evaluation of studies and creation of recommendations.

Level of Evidence	Criteria
**Level 1**	Randomized control trials (including within participants comparison with randomized conditions and crossover designs) without important limitations.
**Level 2**	Randomized control trials with important limitations Observational studies (non-randomized clinical trials or cohort studies) with overwhelming evidence
**Level 3**	Other observational studies (prospective cohort studies, case–control studies, case series)
**Level 4**	Inadequate or no data in population of interest Anecdotal evidence or clinical experience
**Grade of Evidence**	**Criteria**
**Grade A**	Strong recommendation (action can apply to most individuals in most circumstances) Benefits clearly outweigh risks (or vice-versa) Evidence at Level 1, 2, or 3
**Grade B**	Weak recommendation (action may differ depending on individual’s characteristics or other circumstances) Unclear if benefits outweigh risks Evidence at Level 1, 2, or 3
**Grade C**	Consensus recommendation (alternative actions may be equally reasonable) Unclear if benefits outweigh risks Evidence at Level 3 or 4

**Table 3:** Proportion of studies included in systematic review according to level and grade of evidence.

Level of Evidence	Grade	Number of Studies	Proportion
1	A	1	1/26 = 4%
1	B	4	4/26 = 15%
1	C	0	0%
2	A	10	10/26 = 38%
2	B	3	3/26 = 12%
2	C	0	0%
3	A	1	1/26 = 4%
3	B	6	6/26 = 23%
3	C	1	1/26 = 4%
4	C	0	0%

Note: Please refer to Table 2 above for grading scheme (A, B, and C).

**Table 4:** Studies included in the review related to air quality and health risks in patients with coronary artery disease.

Publication	Level/Grade of Evidence	Quality (Out of 15)	CAD Population	Sample Size (Sex)	Mean Age Year ± SD (Range)	Key Findings
**Laboratory Trials**						
Aronow et al. 1972 [31]	2A	8	CAD	10 (100% male)	48 (Range 40–56)	CAD patients experienced significantly increased mean arterial carboxyhemoglobin and mean expired air CO levels during exercise after exposure to heavy traffic **(freeway air)**. ST segment depression was seen in 3 of 10 (30%) of patients. Systolic BP, HR, and forced expiratory volume and forced vital capacity were significantly reduced after breathing freeway air. Angina developed more quickly during exercise after freeway air exposure.
Mills et al. 2007 [32]	1A	10	Previous MI	20 (100% male)	60 ± 1	ST-segment depression was greater during exercise testing when participants were exposed to **diesel exhaust** compared to filtered air. HR (at rest and during exercise) and BP were not significantly different during exposure to diesel exhaust and filtered air. 75% of patients used beta-blockers, no sub-analysis provided.
Mills et al. 2011 [36]	1B	11	CAD	20 CAD (100% male) 32 Healthy (100% male)	CAD = 60 ± 1 (Range 51–67) Healthy = 26 ± 1 (Range 20–38)	Previous MI patients experienced reduced heart rate and HRV (SDNN and TRII) in the 24-h study period post-exposure during and after both clean air and **diesel exhaust** exposures. Therefore, reduced HRV could not be attribute to pollution level. Healthy controls experienced no difference in HRV between 2 and 24-h post-exposure. 75% of MI patients used beta-blockers during the study, however no sub-analysis available.
Routledge et al. 2006 [34]	1B	9	CAD	17 CAD (85% male) 20 Healthy (50% male)	CAD 63 (Range 52–74), Healthy 68 (Range 56–75)	**SO_2_ exposure** led to a decrease in cardiac vagal control (HRV) in healthy participants (observed 4 h after exposure of SO_2_ at **200 ppm**), but not patients with stable angina (HRV, heart rate, or blood pressure). 70% of patients used beta-blockers; sub-analysis not provided for medications (incl. statins and aspirin).
Scaife et al. 2012 [35]	1B	10	Previous MI or coronary bypass grafting patients	18 (78% male)	68 (Range = 56–76)	**Exposure to NO_2_ (400 ppb)** for 1 h produced no significant changes in heart rate, BP, or HRV measures in study patients. Hood delivery system used to ensure other pollutants unlikely the cause of results. No patients in this trial used beta-blockers, though other medications used heterogeneously.
Sheps et al. 1990 [33]	1B 2B	11	Angina pectoris or previous MI or one vessel with 75% stenosis	41 (88% male)	63 (Range = 47–77)	**Exposure to 200 ppm CO** (to induce 6% carboxyhemoglobin) increased the number and complexity of ventricular arrhythmias and the frequency of ventricular premature depolarization in CAD patients within 24 h of exposure. No significant effects were seen in CAD patients when exposed to 100 ppm CO (to induce 4% carboxyhemoglobin). 56% of patients used beta-blockers.
Sinharay et al. 2018 [37]	2A	13	IHD (angiographic evidence)	39 IHD (90% male) 40 healthy (48% male) 40 COPD (48% male)	67 ± 1 IHD 62 ± 1 Healthy 68 ± 1 COPD	Decreased pulse wave velocity and increase augmentation index in non-polluted area following exercise (26-h followup). In a polluted area, with **increased black carbon, NO_2_, PM_10_, PM_2.5_, and ultrafine particles,** pulse wave velocity and augmentation index improvements from exercise were attenuated.
**Real Life Setting**						
Berglind et al. 2009 [6]	2A	9	Previous MI	Augsburg: 1553 (75% male) Barcelona: 941 (80% male) Helsinki: 4025 (54% male) Rome: 7246 (70% male) Stockholm: 11，241 (59% male)	(Range = 35 –>75)	Increased total mortality in MI survivors from five European cities was associated with **PM_10_** and particle number concentrations. When levels were averaged for longer acute periods, (5 and 15 days) **CO and NO_2_** were also associated with mortality.
Chuang et al. 2005 [7]	2A	10	Angina and/or previous MI	10 (68% male)	68.1 ± 3.6 (Range = 61–72)	Decreased SDNN and rMSSD at 2, 3, and 4-h moving averages and 1, 2, and 3-h moving averages, respectively **(PM_0.3-1.0_ exposures)**. Significantly decreased LF at 3 h, and HF at 2 h moving averages (PM_0.3–1.0_). HRV changes were not associated with either PM_1.0–2.5_ or PM_2.5–10_. No patients in this trial used beta-blockers.
Chuang et al. 2008 [39]	2B	10	Angina pectoris or previous MI or worsening stable artery disease	48 (81% male)	57 (Range = 43–75)	At 12 and 24-h average pollution averages, **increased exposure to PM_2.5_, black carbon, and SO_2_** were associated with ST- segment depression in CAD patients (no other HRV measures reported). Beta-blockers did not modify effects of air pollution on ST segment depression. Heterogeneous use of medications, 25% of patients had diabetes.
Dales and Air Pollution-Cardiac Health Research Group (2004) [18]	2A	11	Previous MI	36 (89%)	65 (Range = 51–88)	Patients with CAD not taking beta-blockers had significantly decreased SDNN after exposure to higher levels of **CO**. This effect was not seen after exposure to PM_2.5_. CAD patients taking beta-blockers (25% of subjects) showed no associations.
de Hartog et al. 2009 [19]	2A	8	Angina pectoris or previous MI or percutaneous transluminal coronary angioplasty, or a coronary by-pass surgery	Amsterdam: 33 (66% male), Erfurt: 44 (91% male), Helsinki: 45 (53% male)	Amsterdam: 70.9 (Range = 54–83) Erfurt: 64.3 (Range = 40–78) Helsinki: 68.2 (Range = 54–83)	In patients not taking beta-blockers, PM_2.5_ was associated with decreased SDNN and HF, particularly at longer lag times. **PM_2.5_ from local traffic and long-range transport** most strongly affected HRV (SDNN, and SDNN and HF, respectively) in CAD patients not taking beta-blockers.
Delfino et al. 2010 [17]	2A	10	CAD	64 (59% male)	84 ± 5.6	Increased **organic carbon exposure (from fossil fuel combustion)** positively associated with increases in mean systolic and diastolic BP in elderly participants with CAD. An interquartile increase in organic carbon was associated with 8.2 and 5.8 mmHg in systolic and diastolic BP, respectively (5-day average). Effects were stronger 1–8 h post-reported physical exertion.
Lanki et al. 2006 [40]	2A	8	Angina pectoris or previous MI or percutaneous transluminal coronary angioplasty, or a coronary by-pass surgery	45 (53% male)	68.2 ± 6.5	Examined the relative effects of **fine PM (PM_2.5_)** on exercise-induced ischemia revealing that there are varying effects of PM sources on ST segment depression. PM_2.5_ originating from combustion of long range transport and local traffic sources were most highly associated with ST segment depression during submaximal exercise testing in stable CAD patients.
Lipsett et al. 2006 [9]	2B	9	Angina pectoris or previous MI or percutaneous transluminal coronary angioplasty, or a coronary by-pass surgery	19 (63% male)	71.3 ± 6	Shorter moving average times (up to 8 h) were associated with decrements in SDNN, SDANN, and TRII related to increased exposures to **PM_10_ and PM_10–2.5_**. No effects were seen in HRV after exposure to **PM_2.5_**.
Mirowsky et al. 2017 [41]	3B	11	CAD	13 (100% male)	63 (range = 53–68)	Large artery elasticity index decreased with **increasing ozone exposure**. Ozone was not associated with changes in heart rate variability.
Pekkanen et al. 2002 [38]	3B	8	Angina pectoris or previous MI or percutaneous transluminal coronary angioplasty, or a coronary by-pass surgery	45(53% male)	68.2 ± 6.5	72 out of 342 exercise tests from 45 CAD patients had exercise-induced ST segment depression (>0.1 mV). ST-segment depression associated with **PM_2.5_, PM_1_ NC 0.1–1.0, and UFP 0.01–0.1. NO_2_ and CO** also associated with smaller but increased risk of ST-segment depressions; attributed to exposures 2 days before testing. No ECG changes noted from PM_10–2.5_ exposures. CAD patients not taking beta-blockers showed stronger associations.
Rich et al. 2012 [42]	3B	11	MI or unstable angina	76 (61% male)	Age = *n* (%) <50 = 13 (17) 50–59 = 21 (28) 60–69 = 26 (34) 70–79 = 14 (18) ≥80 = 2 (3)	Adverse changes in SDNN, rMSSD, and systolic blood pressure were associated with increases in at least one of: **ultrafine particles, AMP, and/or PM_2.5_** within a 24-h lag period No significant associations were found in SDNN or mean N-N.
Riojas-Rodriguez et al. 2006 [24]	2A	10	Previous MI	30 (83% male)	55 (NA)	Increased personal exposure by 10 μg/m^3^ to **PM_2.5_** resulted in decreased HF (adjusted for beta-blockers). 1 ppm increase in **CO** had a negative association of LF and VLF; no association with HF. 76% of patients took beta-blockers; 46% exposed to passive smoking
Ruidavets et al. 2005 [43]	2B	10	Previous MI	127 (88% male)	NA	Short-term **O_3_ exposure** (within 1–2 days) was significantly associated with acute MI in middle aged adults (55–64) without history of CAD. No associations were found between acute MI and subjects with history of CAD after exposure to elevated levels of **O_3_, NO_2_ or SO_2_**.
Suh & Zanobetti, 2010 [20]	3B	9	Previous MI	12 (83.3% male)	Male: 59 (NA) Female: 69 (NA)	Personal exposure to **elemental carbon and NO_2_** 24 h prior to HRV measurement was significantly associated to decreased r-MSSD, PNN50, HF and HF/LF ratio. Personal and ambient **exposure to PM_2.5_, and exposure to elemental carbon and NO_2_** was not significantly associated with changes in HRV. Beta-blocker use accounted for in models, but effects not described (small sample size).
Tarkiainen et al. 2003 [21]	3C	8	CAD	6 (100% male)	62 ± 4.4	Elderly CAD patients had significant increases in their rMSSD after **high exposure to CO (4–6 ppm.)**. No other significant changes after CO exposure in HRV measurements were seen.
Von Klot et al. 2005 [10]	2A	9	Previous MI	Augsburg: 60 Barcelona: 61 Helsinki: 68 Rome: 67 Stockholm: 73	Augsburg: 75 Barcelona: 79 Helsinki: 54 Rome: 70 Stockholm: 59	Elevated ambient concentrations of **PM_10_, CO, O_3_, NO_2_, and PNC (estimated for UFP)** were found to be associated with same day hospital cardiac readmissions in MI survivors.
Wheeler et al. 2006 [22]	3B	9	Previous MI	12 (83.3% male)	Male: 59 (NA) Female: 69 (NA)	Interquartile range increase in **NO_2_** were significantly associated with diminished SDNN but not significantly associated with **PM_2.5_**. HR was lower in patients taking beta-blocker medications; SDNN decreased in patients taking β- blockers in response to 4-h ambient PM_2.5_, while patients taking bronchodilators experienced effects in the reverse direction (increased SDNN with PM_2.s_ exposure).
Zanobetti et al. 2010 [23]	3A	9	Angina pectoris or previous MI	46 (80% male)	NA	Increases in **PM_2.5_ and BC** were significantly negatively associated with decreases in HF and rMSSD across all averaging periods (30 min to 120 h). Decreases in SDNN and TP were seen with increased BC at shorter lag periods only (≤2 h). Interactions with medications were not described; >90% of participants were taking beta-blockers. Diagnosis (e.g., history of or current MI) and concurrent conditions (e.g., diabetes) demonstrated different strengths of relationship to exposure, for example diabetics experienced more substantial reductions in rMSSD than non-diabetics.
Zhang et al. 2018 [44]	3B	11	CAD	5332 (61% male)	60 ± 11	Retrospective ECG analyses revealed a short-term (up to 4 days) exposure to air pollution (**ozone and PM_2.5_**) was associated with atrioventricular and intraventricular conduction delays (lengthened PR, QRS, QTc intervals and increasing heart rate), which lasted up to 7 days after exposure.

Abbreviations. AMP: accumulation mode particles (diameter 100–1000 nm); BC: black carbon; BP: blood pressure; CAD: coronary artery disease; CO: carbon monoxide; COPD: chronic obstructive pulmonary disease; ECG: electrocardiogram; h: hour; HR: heart rate; HRV: heart rate variability; HF: high frequency domain (0.15–0.4 Hz); IHD: ischaemic heart disease; LF: low frequency domain (0.04–0.15 Hz); M: males; F: females; MI: myocardial infarction; NA: not available; SO_2_: sulphur dioxide; NO_2_: nitrogen dioxide; O_3_: ozone; PM_10_: the mass concentration of course particles with aerodynamic diameters of <10 μm; PM_2.5_: the mass concentration of fine particles with aerodynamic diameters of <2.5 μm; PM_0.3–1.0_: particulate matter with aerodynamic diameters between 0.3 and 1.0 μm; PNC: particle number concentration; PNN50: percent of absolute differences between successive normal R-R intervals that exceed 50 ms; QRS complex: ventricular depolarization on electrocardiogram (ECG); QTc: corrected QT interval; rMSSD: square root of the mean of the sum of squares of successive differences between adjacent N-N intervals; SDNN: standard deviation of normal-to-normal (N-N) intervals; SDANN: standard deviation of average normal-to-normal (N-N) intervals within successive 5-min blocks; ST segment: portion of ECG from ventricular depolarization to repolarization; TP: total power; TRII: triangular index; UFP: ultrafine particle; VLF: very low frequency domain (0.0033 to 0.04.H). Bold font indicates air pollution exposures in each study. SD: standard deviation. Note: Bolded text is used to highlight the air pollutant exposure in each study.

## Supplementary Materials

The following are available online at https://www.mdpi.com/2077-0383/8/2/274/s1, Figure S1: The Air Quality Health Index (AQHI) and related health messages from Health Canada and Environment Canada.
