# Supplementary material for: A Systematic Review of the Short-Term Health Effects of Air Pollution in Persons Living with Coronary Heart Disease
Source: J Clin Med. 2019 Feb 24;8(2):274. doi: 10.3390/jcm8020274 (PMC6406357; doi:10.3390/jcm8020274)
Supplement: Supplementary file 1 [file jcm-08-00274-s001.pdf]

Supplementary Information

Supplementary Figure S1: The Air Quality Health Index (AQHI) and related health messages from Health Canada and Environment Canada.

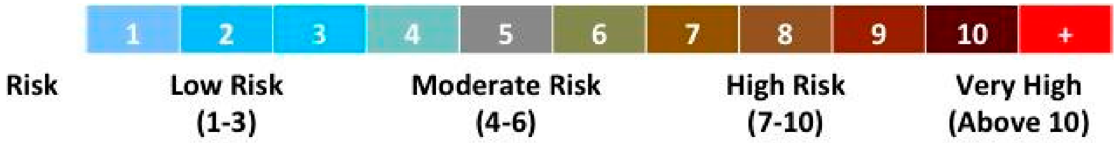

Health messages for "at risk" individuals and the general public for each of the AQHI Health Risk Categories.

**Health Risk**	**Air Quality Health Index**	**Health Messages**	
		**At Risk Population**[1]	**General Population**
Low	1 - 3	Enjoy your usual outdoor activities.	Ideal air quality for outdoor activities.
Moderate	4 - 6	Consider reducing or rescheduling strenuous activities outdoors if you are experiencing symptoms.	No need to modify your usual outdoor activities unless you experience symptoms such as coughing and throat irritation.
High	7 - 10	Reduce or reschedule strenuous activities outdoors. Children and the elderly should also take it easy.	Consider reducing or rescheduling strenuous activities outdoors if you experience symptoms such as coughing and throat irritation.
Very High	Above 10	Avoid strenuous activities outdoors. Children and the elderly should also avoid outdoor physical exertion.	Reduce or reschedule strenuous activities outdoors, especially if you experience symptoms such as coughing and throat irritation.

[1] People with heart or breathing problems are at greater risk. Follow your doctor's usual advice about exercising and managing your condition. Table adapted from information provided by Health Canada and Environment Canada (http://www.ec.gc.ca/cas-aqhi/).
